# HIF1 driven transcriptional activity regulates steroidogenesis and proliferation of bovine granulosa cells

**DOI:** 10.1038/s41598-020-60935-1

**Published:** 2020-03-03

**Authors:** Vijay Simha Baddela, Arpna Sharma, Marten Michaelis, Jens Vanselow

**Affiliations:** 1 Experimental Reproductive Biology Unit, Institute of Reproductive Biology, Leibniz Institute for Farm Animal Biology (FBN), 18196 Dummerstorf, Germany; 2 Reproductive Biochemistry Unit, Institute of Reproductive Biology, Leibniz Institute for Farm Animal Biology (FBN), 18196 Dummerstorf, Germany

**Keywords:** Cell biology, Molecular biology

## Abstract

Hypoxia-inducible factor 1 (HIF1) is a heterodimeric transcription factor, consisting of a constitutively expressed β-subunit (HIF1B) and a regulated α-subunit (HIF1A). In the present study, we analyzed the HIF1 driven transcriptional activity in bovine granulosa cells (GC). Treatment of GC with FSH (follicle stimulating hormone) and IGF1 (insulin-like growth factor 1) resulted in the upregulation of *HIF1A* mRNA expression under normoxia. Immunohistochemistry of bovine ovarian sections showed distinct staining of HIF1A in the GC layer of different staged ovarian follicles. Suppression of HIF1 using echinomycin and gene knockdown procedures revealed that HIF1 transcriptionally regulates the genes associated with steroidogenesis (*STAR*, *HSD3B* and *CYP19A1*) and proliferation (*CCND2* and *PCNA*) of GC. Further, our data suggest that *CYP19A1*, the key gene of estradiol production, is one of the plausible downstream targets of HIF1 in bovine GC as shown by gene expression, radioimmunoassay, and chromatin precipitation analysis. Based on these results, we propose that HIF1 driven transcriptional activity plays a crucial role in GC functionality, especially steroidogenesis and proliferation in developing bovine ovarian follicles.

## Introduction

Hypoxia-inducible factor 1 is a pleiotropic transcription factor made up of hypoxia-inducible factor 1 A (HIF1A) and hypoxia-inducible factor 1B (HIF1B). HIF1A is the specific and regulatory constituent of HIF1 protein complex, whereas HIF1B is the constitutive subunit and forms quaternary structures with multiple other proteins such as ligand-bound aryl hydrocarbon receptor, aryl hydrocarbon receptor repressor, and HIF2A^1^. Therefore regulation of *HIF1A* expression is unique and vital to the activity of the HIF1 protein complex. HIF1 binds to hypoxia-responsive elements (HRE) present in the promoter region of target genes thus controlling their transcription. The groundbreaking investigations have revealed that HIF1 is a master transcriptional regulator of cellular response to low oxygen levels^2–4^. However, *HIF1A* is also induced and stabilized in hypoxia independent manner by different growth factors such as angiotensin II^5^, prostaglandins^6^, interferon-alpha^7^, insulin-like growth factor 1 (IGF1)^8^ etc. It has been implied that Wnt induced phosphatidylinositol 3-kinase (PI3k)/Akt signaling and signal transducers and activators of transcription 3 (STAT3), and c-Myc pathways can induce the activity of HIF1 in a hypoxia independent manner^9^.

Emerging studies indicate that HIF1 is a significant regulator of gene expression in ovarian compartments, and play a role in healthy follicle development. Transcriptome analysis in pigs indicated that *HIF1A* expression is downregulated in atretic follicles compared to medium sized healthy antral follicles^10^. Expression of *HIF1A* was reported in granulosa cells (GC) of different species including human^11^, mice^12^, rat^13^, pigs^14^ and cows^15^. Kim *et al*. (2009) have shown that HIF1 is essential for ovulation process as inhibition of HIF1 activity using echinomycin, a competitive inhibitor of HIF1, caused an anovulatory phenotype in mice^12^.

HIF1 regulates multiple genes in GC and contributes to the ovarian follicle development and differentiation. Zhang *et al*. (2015) have shown that knockdown of *HIF1A* mRNA abundance downregulates the *PCNA* (proliferator cell nuclear antigen) mRNA expression under normoxic conditions in rat primary GC^13^, similar to renal medullary interstitial cells of rats^5^. Alam *et al*. (2004) reported that FSH (follicle stimulating hormone) mediated upregulation of genes involved in follicle differentiation such as *VEGFA* (vascular endothelial growth factor A), *LHCGR* (Luteinizing Hormone/Choriogonadotropin Receptor) and *INHBA* (inhibin-α) is dependent on HIF1 activity in rats^16^. Another gene, *END2* (endothelin 2), which is suggested to play a role in ovulation and luteinization processes, was found to be regulated by HIF1 in transformed human GC^17^. Similarly, *STAR* coding for steroidogenic acute regulatory protein was transcriptionally regulated by HIF1 both under normoxic and hypoxic conditions in KK1 cells, which are immortalized mouse GC^18^.

It is well known that vascularization of the ovarian follicle is limited to the thecal cell layer, which is separated from the GC and cumulus-oocyte complex (COC) by a basement membrane. Therefore, it has been implied that significantly lower amounts of oxygen will be available to the intrafollicular cells as the follicle’s size increases^19^. Hence, analyzing the role of HIF1 under normoxic and hypoxic environments in the presence of FSH and IGF1 would offer important cues regarding GC physiology. Accordingly, the present investigation was carried out to identify HIF1 dependent transcriptional activity both under normoxic and hypoxic conditions using our renowned estrogen active culture model of bovine primary GC^20–23^.

## Results

### Expression of HIF1A in bovine granulosa cells

**Figure 1:**
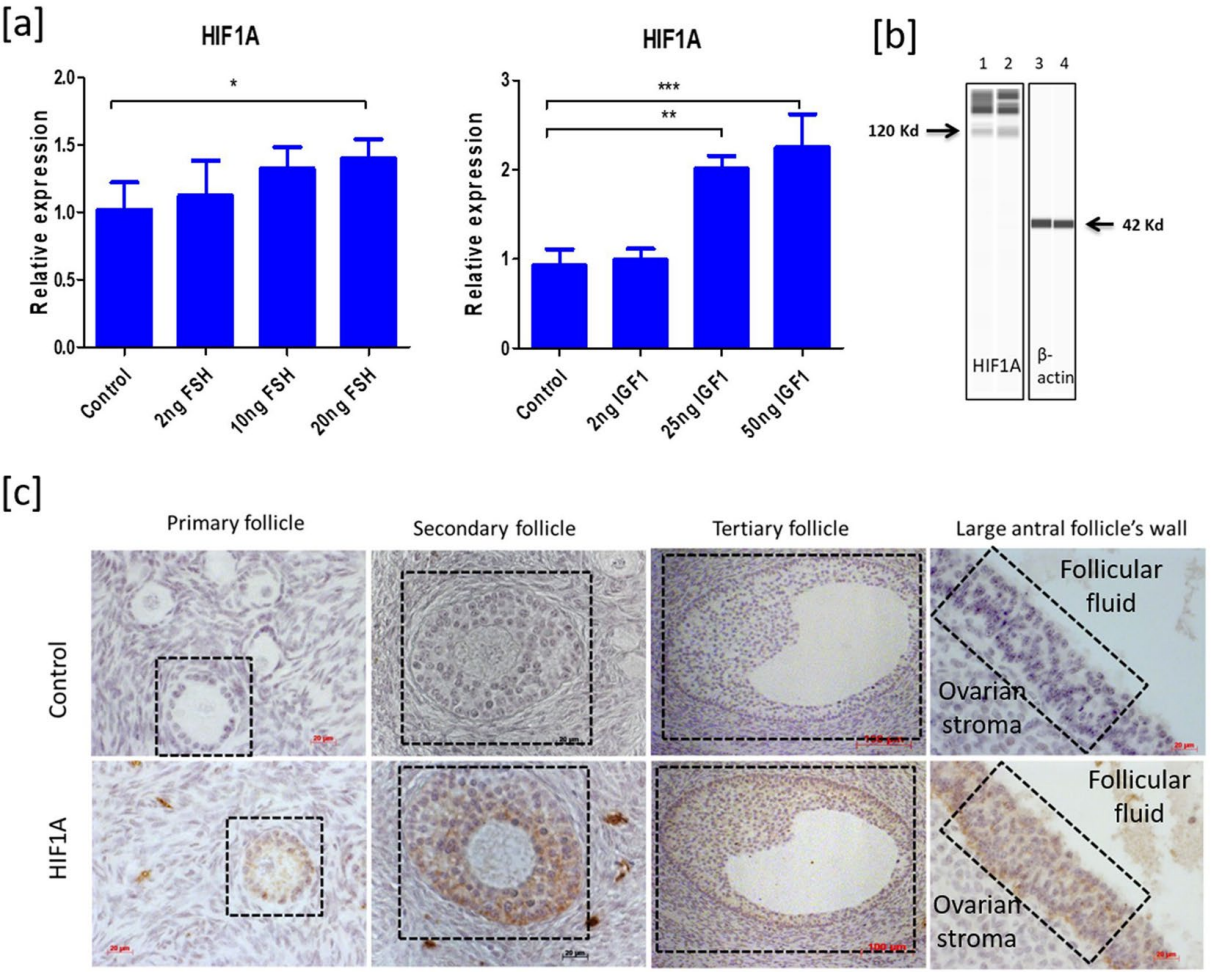
Expression analysis of HIF1A in granulosa cells. (**a**) indicates the mRNA expression of HIF1A under different FSH and IGF1 concentrations. (**b**) Indicates the detection of HIF1A protein under normoxia. The lane numbers 1–4 indicate the western runs of individual protein lysates. Columns 1 and 2 represent duplicates of HIF1A probing in FSH (20 ng/ml) and IGF1(50 ng/ml) treated GC under normoxia while columns 3 and 4 represent the Beta actin (BACT) probing in the corresponding samples. The arrow marks in (**b**) indicate the HIF1A (columns 1 and 2) and BACT (columns 3 and 4) protein bands. The image is obtained with the exposure time of 8 seconds in Simple Western instrument. (**c**) illustrates the immunolocalization of HIF1A in different growing ovarian follicles of abattoir ovaries. The brown color immunochemical signal indicates the HIF1A staining inside the follicle. 400x optical magnification was used for primary and secondary and large antral follicle’s wall. 100x optical magnification was used for tertiary follicles in the pictures. Inside dotted box indicates the specific follicle for primary, secondary and tertiary follicle sections whereas dotted box points the GC layer in large antral follicle’s wall. Data were presented in Mean ± SEM values of three independent cell culture experiments (n = 3) for Fig. 1a. Significant differences were acknowledged at the minimum level of p < 0.05 by one way repeated measures analysis of variance. Pairwise comparisons were analyzed using Post hoc Tuckey test.

The effect of FSH was analyzed at three different concentrations, such as 2 ng/ml, 10 ng/ml and 20 ng/ml (Fig. 1a). At 2 and 10 ng ml FSH, the expression of *HIF1A* was not altered in GC. However, *HIF1A* was significantly induced at 20 ng/ml FSH compared to the control group (0 ng FSH and 0 ng IGF1). Likewise, the effect of IGF1 was analyzed at concentrations of 2 ng/ml, 25 ng/ml and 50 ng/ml (Fig. 1a). Similar to FSH, IGF1 was unable to induce *HIF1A* at the lowest concentration. However, the expression of *HIF1A* was profoundly increased at 25 ng/ml and 50 ng/ml. No difference in the *HIF1A* expression was observed between 25 ng/ml and 50 ng/ml IGF1 treatments. The western probing analysis showed that FSH (20 ng/ml) and IGF1 (50 ng/ml) supplemented GC synthesize HIF1A protein under normoxia (Fig. 1b). Immunohistochemistry of bovine ovarian follicles revealed that HIF1A proteins are distinctly expressed in the GC layer of primary, secondary, tertiary, and large antral follicles, which are in general under the influence of FSH and IGF1 *in-vivo* (Fig. 1c).

### Effects of echinomycin on granulosa cells

**Figure 2:**
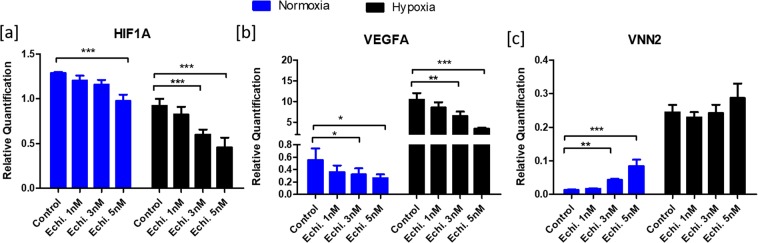
Effects of echinomycin on candidate gene expression. Suppression of HIF1 activity using echinomycin altered the mRNA expression of *HIF1A* (**a**), *VEGFA* (**b**) and *VNN2* (**c**) genes under normoxic and hypoxic conditions. Data were presented in Mean ± SEM values of three independent cell culture experiments (n = 3). Significant differences were acknowledged at the minimum level of p < 0.05 by one way repeated measures analysis of variance. Pairwise comparisons were analyzed using Post hoc Tuckey test.

Initially, effect of echinomycin on cell GC viability was analyzed to primarily to avoid cytotoxic doses of echinomycin in GC cultures. Results showed that 5 nM of echinomycin has no significant effect on cell viability, apoptosis and dead cell status compared to the control cells treated with 20 ng/ml FSH and 50 ng/ml IGF1 without echinomycin (Supplementary Information: S3,S4). In subsequent analyses, the expression of *HIF1A* (Fig. 2a) was decreased under normoxic and hypoxic conditions upon echinomycin treatment. Echinomycin significantly downregulated the expression of *VEGFA* (Fig. 2b), which is a well-known downstream target of HIF1 in many cell types, including GC. Therefore, the decrease in *VEGFA* expression reflects the successful inhibition of HIF1 activity by echinomycin both under normoxic and hypoxic conditions. On the contrary, inhibition of HIF1 function caused an increased expression of an inflammatory gene, vascular non-inflammatory molecule 2 (*VNN2*) (Fig. 2c).

#### Steroidogenesis

**Figure 3:**
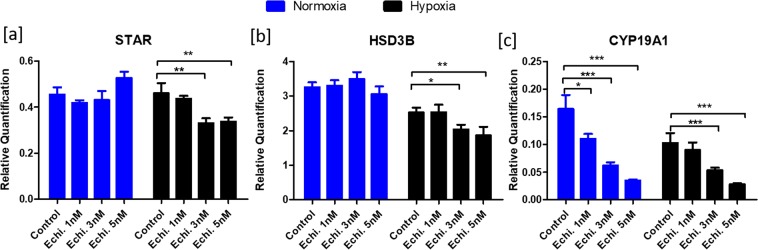
Effects of echinomycin on the expression of steroidogenic genes. Figure depicts the normalized mRNA expression values of *STAR* (**a**), *HSD3B* (**b**) and *CYP19A1* (**c**) genes under normoxic and hypoxic conditions. Data were presented in Mean ± SEM values of three independent cell culture experiments (n = 3). Significant differences were acknowledged at the minimum level of p < 0.05 by one way repeated measures analysis of variance. Pairwise comparisons were analyzed using Post hoc Tuckey test.

Echinomycin did not affect the *STAR* and *HSD3B* (3 beta hydroxy delta 5 steroid dehydrogenase) mRNA expression at normoxia but decreased their expression under hypoxia at 3 nM and 5 nM concentrations (Fig. 3a,b). Interestingly, echinomycin caused a dose-dependent downregulation of cytochrome P450 Family 19 Subfamily A Member 1 (*CYP19A1*; Fig. 3c) expression under both oxygen conditions.

#### Cell Proliferation

**Figure 4:**
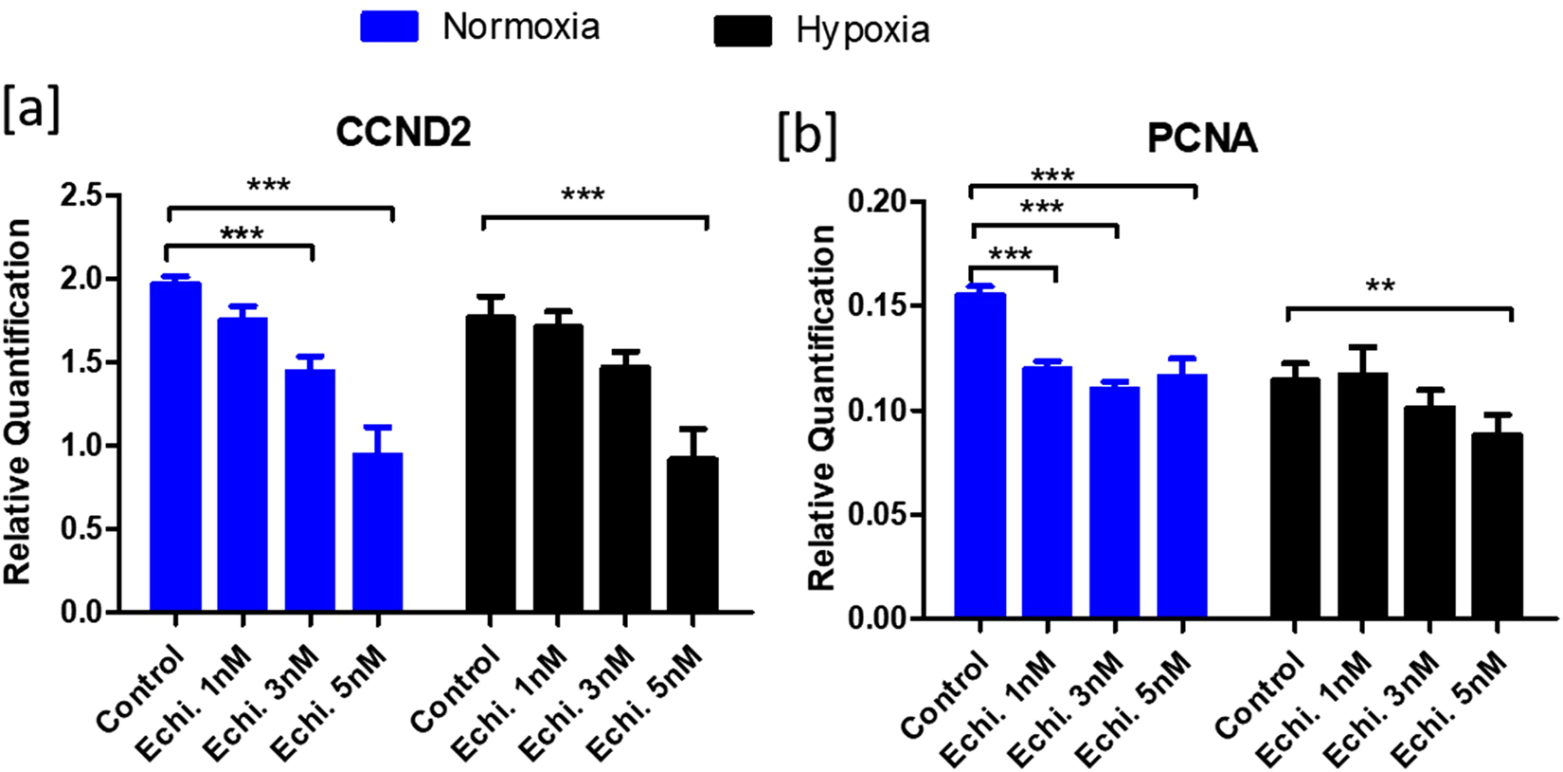
Effects of echinomycin on cell proliferation markers: Figure depicts the normalized mRNA expression data of *CCND2* (**a**) and *PCNA* (**b**) genes upon inhibition of HIF1 activity using echinomycin. Data were presented in Mean ± SEM values of three independent cell culture experiments (n = 3). Significant differences were acknowledged at the minimum level of p < 0.05 by one way repeated measures analysis of variance. Pairwise comparisons were analyzed using Post hoc Tuckey test.

Expression of cyclin D2 (*CCND2*) and *PCNA* were analyzed to determine the effect of HIF1 on cell proliferation. Echinomycin mediated inhibition of HIF1 function resulted in the downregulation of *CCND2* (Fig. 4a) and *PCNA* (Fig. 4b) under both normoxic and hypoxic conditions.

### Effects of HIF1A knockdown in granulosa cells

**Figure 5:**
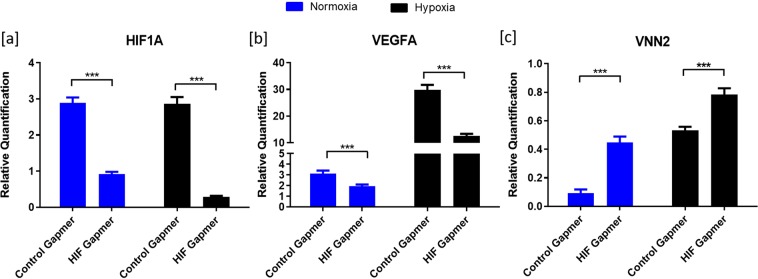
Effects of HIF1A knockdown on candidate gene expression. Figure depicts the mRNA expression values of *HIF1A* (**a**), *VEGFA* (**b**) and *VNN2* (**c**) genes upon *HIF1A* knockdown under normoxic and hypoxic conditions. Data were presented in Mean ± SEM values of three independent cell culture experiments (n = 3). Significant differences were acknowledged at the minimum level of p < 0.05 by t-test.

GC health was unaffected by the *HIF1A* gapmer at 50 nM concentration as there were no significant changes in the viability, apoptosis, and dead cell status (Supplementary Information: S5,S6). qPCR quantification showed that *HIF1A* mRNA expression was significantly knocked down under normoxic and hypoxic conditions by the *HIF1A* gapmers (Fig. 5a). Similar to the echinomycin treatment, expression of *VEGFA* was downregulated (Fig. 5b) and *VNN2* was upregulated upon *HIF1A* knockdown (Fig. 5c).

#### Steroidogenesis

**Figure 6:**
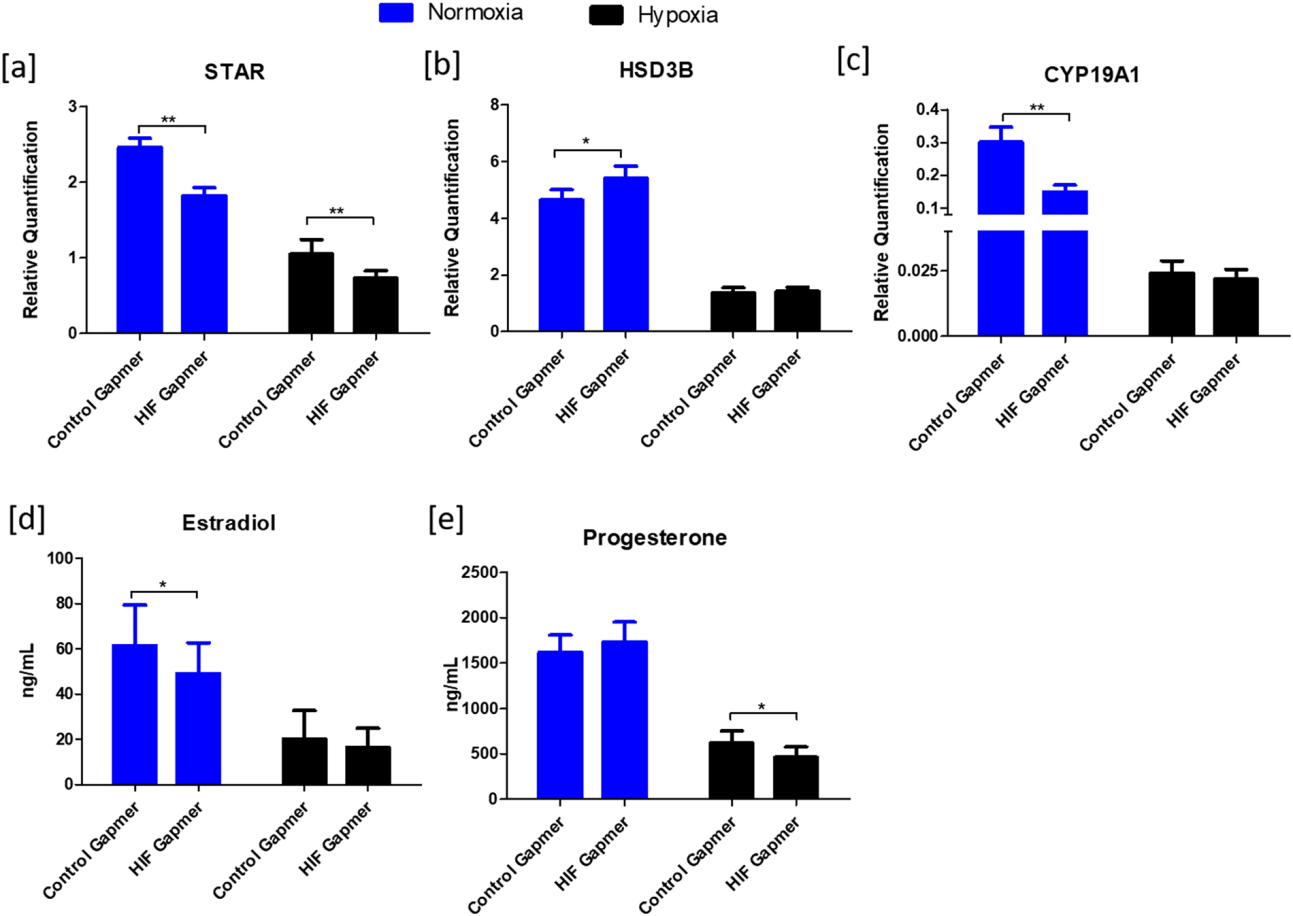
Effects of HIF1A knockdown on the expression of steroidogenic genes and steroidogenesis. Suppression of HIF1 expression by knockdown altered *STAR* (**a**), *HSD3B* (**b**) and *CYP19A1* (**c**) transcript abundance. (**d**,**e**) show the estradiol and progesterone concentrations in the spent culture media. Data were presented in Mean ± SEM values of three independent cell culture experiments (n = 3). Significant differences were acknowledged at the minimum level of p < 0.05 by t-test.

Suppression of *HIF1A* expression caused a consistent downregulation of *STAR* expression both under normoxic and hypoxic conditions (Fig. 6a). A marginal yet significant upregulation of *HSD3B* expression was detected at normoxia (Fig. 6b), but no changes have been observed under hypoxia. *CYP19A1* expression was significantly downregulated at normoxia (Fig. 6c) while no such effects were observed at hypoxia with *HIF1A* knockdown. Estimation of steroids using radioimmunoassay specified a marginal yet significant decrease in the production of estradiol at normoxia and progesterone at hypoxia (Fig. 6d,e) upon knocking down *HIF1A* expression. The effects were not significant concerning estradiol and progesterone production under hypoxic and normoxic conditions, respectively.

#### Cell proliferation

**Figure 7:**
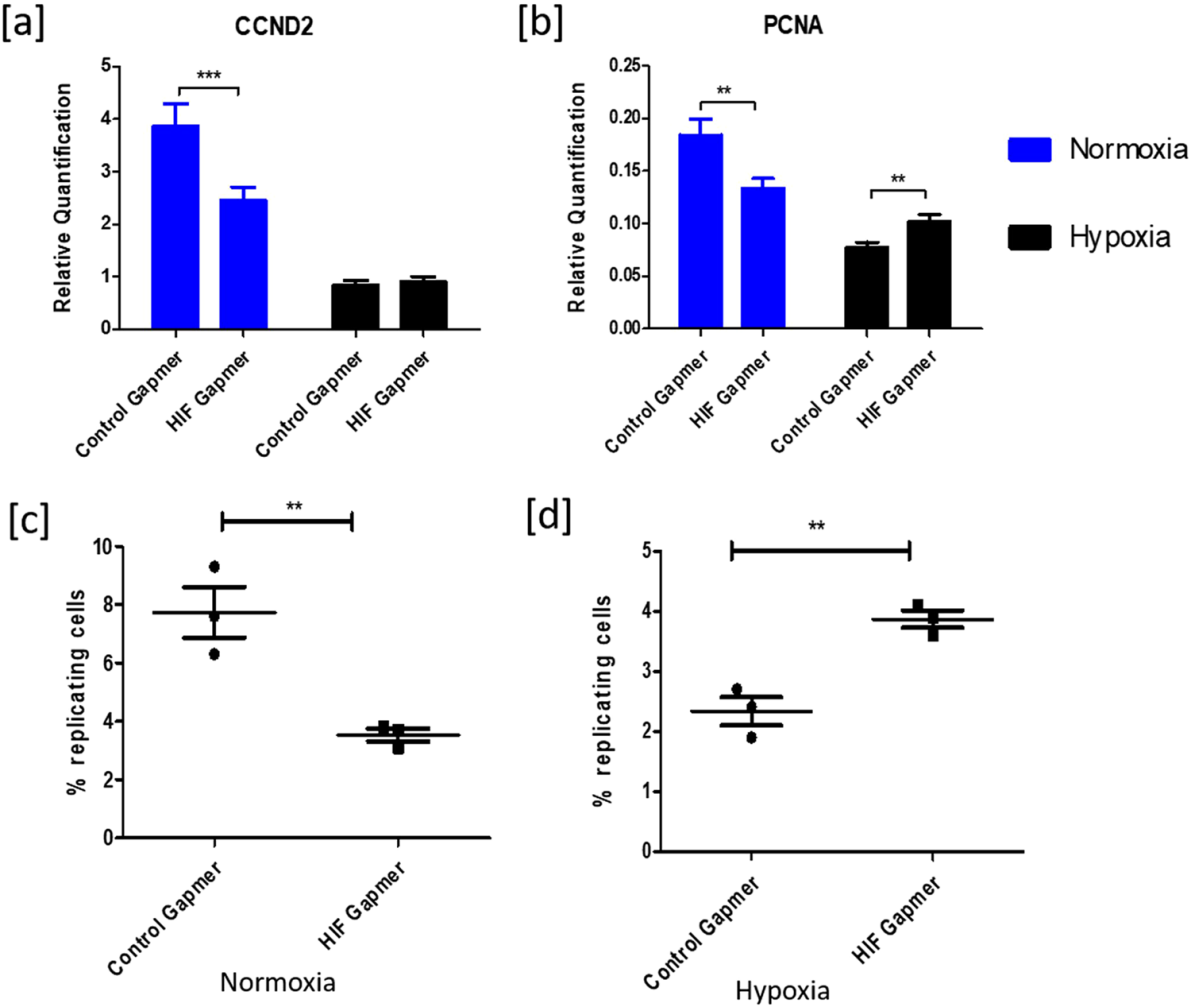
Effects of HIF1A knockdown on cell proliferation. (**a**,**b**) depicts CCND2 and PCNA mRNA expressions upon HIF1A knockdown in granulosa cells under normoxic and hypoxic conditions. (**c**,**d**) depict the percent of replicating cells (S-phase) under normoxic and hypoxic conditions, respectively, upon treating with control and HIF1A gapmers. Data were represented in the mean ± SEM values of three independent culture experiments (n = 3). Significant differences were acknowledged at the minimum level of p < 0.05 by t-test.

Similar to the effects of echinomycin, *HIF1A* knockdown induces downregulation of cell proliferation markers *CCND2* and *PCNA* under normoxic conditions (Fig. 7a,b). However, *PCNA* expression was increased upon *HIF1A* knockdown at hypoxia. These effects were further analyzed using flow cytometry, which indicated that knockdown of *HIF1A* leads to decreased and increased numbers of cells in DNA replication stage under normoxic (Fig. 7c) and hypoxic (Fig. 7d) conditions, respectively.

### Chromatin immunoprecipitation analysis

**Figure 8:**
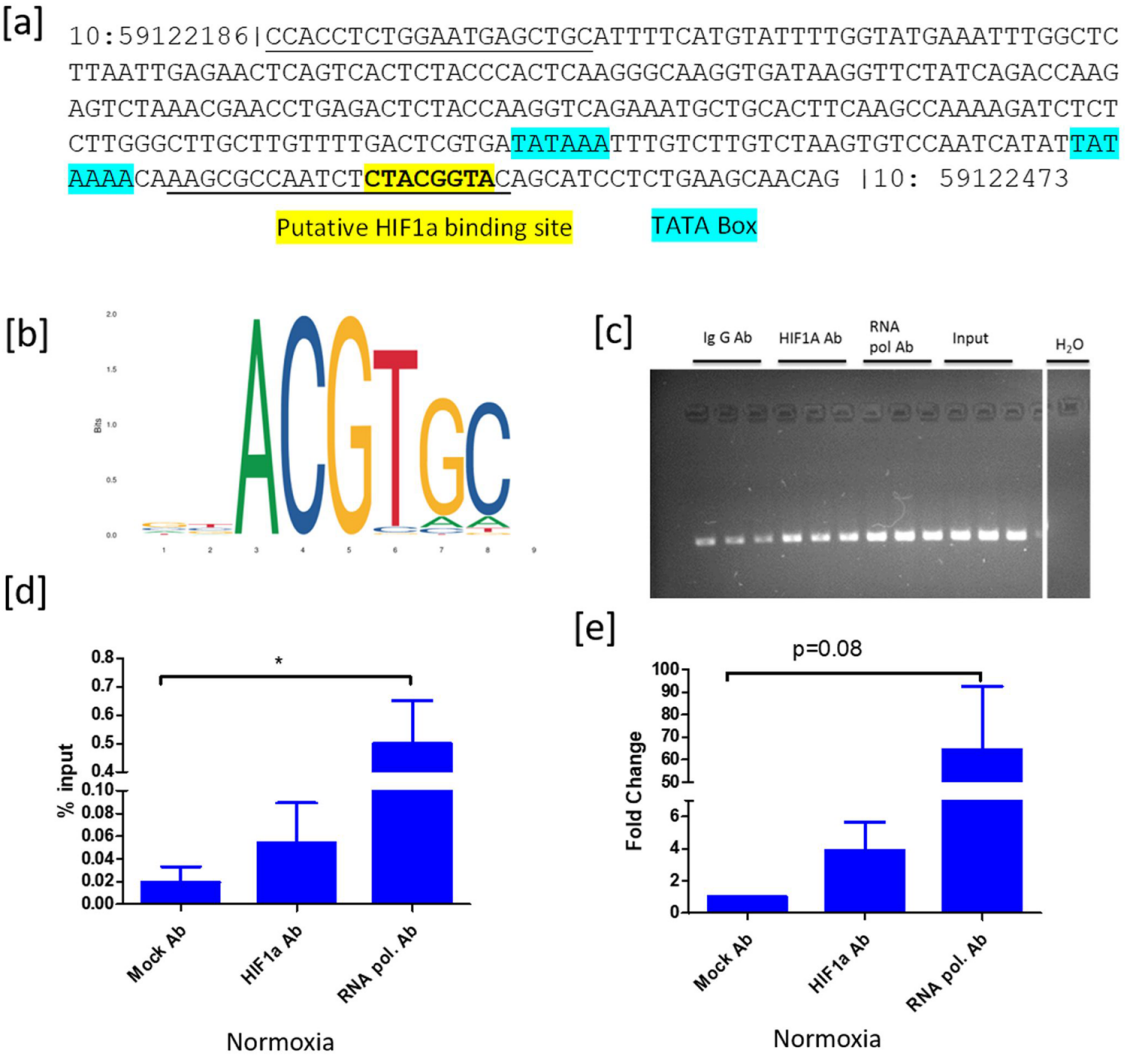
Chromatin immunoprecipitation analysis. (**a**) shows the nucleotide sequence between the physical location of 59122186 to 59122473 nucleotides on chromosome 10 in the bovine genome. Manually curated TATA box and the putative HIF1 binding sites were highlighted in blue and yellow colors. The primer binding sites were indicated with an underline. (**b**) adopted JASPAR Sequence logo of the HIF1 binding site based on ChIP seq data in humans. (**c**) indicates the agarose gel pictures of immune-precipitated and input DNA samples. (**d**,**e**) depict the relative quantification of HIF1 binding events on the CYP19A1 promoter based on percent input and fold change methods, respectively under FSH and IGF1 Supplemented conditions. Data were represented in mean ± SEM values of three independent experiments (n = 3). Significant differences were acknowledged at the minimum level of p < 0.05 by one way repeated measures analysis of variance. Pairwise comparisons were analyzed using Post hoc Tuckey test.

*CYP19A1* showed a consistent and robust downregulation both in echinomycin and knockdown studies upon affecting HIF1 activity and expression. Sequence analysis of the *CYP19A1* proximal promoter P2 (Fig. 8a) indicates the availability of a putative HIF1 binding site, which is recognized based on the conserved HIF1 binding sequence reported in JASPER database (http://jaspar.genereg.net) (Fig. 8b), similar to human CYP19A1 promoter^24^. Therefore, we analyzed the binding of HIF1A in the bovine *CYP19A1* proximal promoter region to derive possible cues on the regulation of *CYP19A1* expression by HIF1 under FSH and IGF1 stimulated conditions. The results indicate the binding of HIF1 transcription factor in the *CYP19A1* promoter region, which can be seen in the agarose gel images of PCR products (Fig. 8c). Further, substantial binding of anti RNA polymerase antibody to the *CYP19A1* promoter indicates an active transcription of the *CYP19A1* gene in granulosa cells under FSH and IGF1 treated conditions (Fig. 8d,e).

## Discussion

The present study is carried out understand the transcriptional activity of HIF1 in GC isolated and cultured from the small antral follicles. It is well known that FSH and IGF1 induced steroidogenesis and proliferation of GC is obligatory for ovarian follicle development in many mammals, including cows^25,26^. Therefore supplementation of FSH and IGF1 is universally practiced for GC cultures, especially for attaining an “estradiol active” phenotype^27–31^. In the present study, treatment of GC with physiologically relevant doses of FSH (20 ng/ml) and IGF1 (25 and 50 ng/ml) independently induced the mRNA expression of *HIF1A*. Therefore, *HIF1A* levels reported in developing ovarian follicles^14,15,32^ and in the cultured GC^22^ could be because of the actions of FSH and IGF1 signaling. However, other endocrine factors such as pregnant mare serum gonadotropins also shown to induce HIF1A expression in ovarian follicle^13^, suggesting the physiological state of the animals plays a role in HIF1A expression in ovary.

In the present study, the loss of HIF1 function in GC was studied using echinomycin and *HIF1A* gene knockdown approaches under normoxic and hypoxic conditions. Echinomycin competitively inhibits the transcription factor activity of HIF1 by binding to hypoxia responsive elements (HRE) on the DNA. Surprisingly, mRNA expression of *HIF1A* was downregulated upon echinomycin treatment. This might be because several miRNAs as miR-200, miR-153-3p, miR-429, and miR-18a have been reported to suppress the gene expression of HIF1A in different cells types^33–36^. On the other hand, Shen *et al*. (2013) have indicated that HIF1 stabilization could inhibit miR-200b expression^37^. Therefore, it might be possible that echinomycin mediated suppression of HIF1 activity may enable the expression of miR-200 and of other miRNAs that could target *HIF1A* mRNA. A similar lower expression of *HIF1A* was observed under hypoxic conditions compared to normoxia. HIF1A protein is well known to have less stability under normoxic conditions. Whereas under hypoxic conditions, HIF1A protein is stabilized due to inhibition of prolyl hydroxylase activity^2,38^. Therefore we speculate that the HIF1A mRNA expression under hypoxic conditions might also be subjected to a feedback regulation compared to the normoxic conditions and result in a bit lower expression in hypoxia. In any case, further dedicated experiments are needed to understand the regulation of transcript abundance of *HIF1A* in GC under normoxic and hypoxic conditions and echinomycin treatment.

Loss of HIF1 function consistently resulted in the downregulation of *VEGFA*, which is a well-established downstream effector of *HIF1A* action in different cell types. Most importantly, this particular effect on *VEGFA* expression confirms the suppression of HIF1 activity in GC after the treatment with echinomycin and anti-*HIF1A* gapmer and also indicates the functionality of HIF1 both at normoxic and hypoxic environments. A similar finding regarding FSH induced expression of *VEGFA* in GC is shown to be mediated via HIF1 in mice^39^. In the present study, VEGFA has shown a lower expression in normoxia compared to hypoxia possibly because of the lower stability of HIF1A protein under normoxic conditions compared to hypoxia. However, it is possible that *VEGFA* expression could also depend on other companion factors such as activator protein 1 (AP1) and specificity protein 1 (SP1)^40^, whose expression was not analyzed in the present investigation.

*VNN2* has been reported to be involved in inflammatory processes by inducing leukocyte migration and adhesion processes in physiological conditions^41^. We have analyzed the expression of *VNN2* as it was shown to be upregulated in GC isolated from pre-ovulatory follicles after LH surge^42^, which are implied to be on the edge of severe hypoxic conditions^19^. Our data revealed that the expression of *VNN2* is upregulated by hypoxia in GC. But knockdown of *HIF1A* mRNA abundance downregulated the expression of *VNN2* both under normoxic and hypoxic conditions. This important observation suggests that the upregulation of *VNN2* in GC of the ovulatory follicle maybe because of the prevailing hypoxic conditions rather than actions of LH. Importantly, the inhibitory action of HIF1 on *VNN2* suggests its anti-inflammatory actions under FSH and IGF1 Supplemented conditions^43^.

The expression of three key steroidogenic genes (*STAR*, *HSD3B*, and *CYP19A1*) was analyzed upon inhibiting the HIF1 function. *STAR* catalyzes a rate-limiting process of transporting cholesterol from the outer into the inner mitochondrial membrane in steroidogenic cells. *HSD3B* catalyzes the conversion of pregnenolone into progesterone. The *CYP19A1* coded aromatase converts androgens into estrogens, which are essential female sex hormones^44^. Regulation of these three steroidogenic genes by *HIF1A* appears to be dynamic and tissue-dependent as revealed by different studies. The expression of STAR was shown to be induced by HIF1 in mouse KK1 cells^18^. Conversely, the inhibitory action of HIF1 on *STAR* expression is documented in mouse Leydig cells^45^. Similar reports can be found that HIF1 could increase *HSD3B* expression in Leydig cells^46^, while others suggest that it may inhibit *HSD3B* and progesterone production in canine luteal cells^47^. Likewise, HIF1 has been shown to induce *CYP19A1* expression in breast adipose stromal cells^24^ and inhibits the same in H295R cells, which are steroidogenic adrenal cortical cells^48^. In the present study, we could show that inhibition of HIF1 function leads to dose-dependent suppression of *CYP19A1* expression and estradiol production in bovine GC under normoxic conditions. Indeed, a chromatin precipitation analysis indicated significant binding of HIF1 to the *CYP19A1* proximal promoter in breast adipose stromal cells^24^. Our results are in agreement with this earlier study as we could see the binding of *HIF1* on *CYP19A1* proximal promoter in chromatin precipitation analysis. Therefore, the present results suggest that *CYP19A*1 is a plausible HIF1 target gene in granulosa cells of developing follicles, especially under FSH and IGF1 signaling conditions. However, this stimulatory effect of HIF1 on *CYP19A1* expression and estradiol production was not seen under hypoxic conditions upon *HIF1A* knockdown possibly because of the attenuation of FSH and IGF1 signaling. HIF1 has been shown to be involved in the hypoxic synthesis of progesterone in GC^49^. Our results are in the scope of this finding as inhibition of HIF1 activity decreased the progesterone production at hypoxia by possibly inhibiting *STAR* and *HSD3B* expression. We observed an apparent discrepancy between echinomycin and gapmer treatments concerning *STAR* and *HSD3B* gene expression. Echinomycin did not alter STAR expression under normoxia but could significantly decrease the same under hypoxia. In contrast, *HIF1A* knockdown consistently decreased *STAR* expression both under normoxic and hypoxic conditions similar to murine GC^18^.

*PCNA* and *CCND2* are well known positive markers of cell proliferation in many cells. In the ovary, both genes might play an essential role in follicular maturation as the proliferation of GC is an essential feature of follicular growth and development^13^. Moreover, *CCND2* and *PCNA* have been used in a number of studies to mark the proliferation status of GC *in-vitro* and *in-vivo*^42,50,51^. Expression of *PCNA* and *CCND2* was downregulated at normoxia and hypoxia in the presence of either echinomycin or *HIF1A* gapmers, indicating the contribution of HIF1 in GC proliferation under normoxic conditions. However, the effects of echinomycin on these genes appeared to be more prominent at all tested concentrations in normoxia than hypoxia likely because of the higher proliferation of GC under normal oxygen levels^22^. Most importantly, these results indicate the active role of HIF1 in FSH and IGF1 induced proliferation of GC. A similar downregulation of *PCNA* expression upon HIF1 inhibition has been documented in rat GC under normoxic conditions^13^. On the other hand, it has been shown that HIF1 induces cell cycle arrest at hypoxia in different cell types^52,53^. Similarly, we could observe an upregulation of *PCNA* gene expression at hypoxia following knockdown of *HIF1A* expression. However, similar to *STAR* and *HSD3B* genes we did not see this similar regulation in echinomycin treatment. In any case, it might be important to acknowledge the plausible off-target effects induced by chemical inhibitors under different circumstances^54^. Fluorescence-activated cell sorting (FACS) analysis has further confirmed that HIF1 knockdown could induce cell cycle progression under hypoxia. Based on these data, we could interpret that HIF1 conversely regulates cellular proliferation between normoxic and hypoxic conditions. To the best of our knowledge, this is the first report to show differential regulation of cell proliferation by HIF1 under normoxic and hypoxic conditions. We further speculate that the function of HIF1 might be dependent upon the original stimulus that induces HIF1 function. For instance, FSH and IGF1 induced HIF1 function at normoxia is associated with GC proliferation together with other companion signaling molecules. If the stimulus is hypoxia, HIF1 might be involved in the suppression of cell proliferation^55^ as cells do not have an adequate supply of energy resources such as ATP under hypoxic conditions. These novel insights into HIF1 function could be valuable to understand that differential modulation of the activities of effector molecules depends on the initial stimulus. However, future omics based investigations are needed to further understand the differential role of HIF1 under both normoxic and hypoxic conditions.

Overall, the present study provides a molecular cue regarding the transcriptional regulation of HIF1 in GC both under normoxic and hypoxic conditions. We want to acknowledge that these results may represent the regulation by HIF1 in developing follicle where the GC show an estrogen active phenotype. We also acknowledge that the results under hypoxia are not representative of the situation in matured preovulatory follicles as we did not treat the cells with LH. However the data provide interesting and novel clues regarding the regulation of genes in GC by HIF1 under hypoxia.

## Conclusions

**Figure 9:**
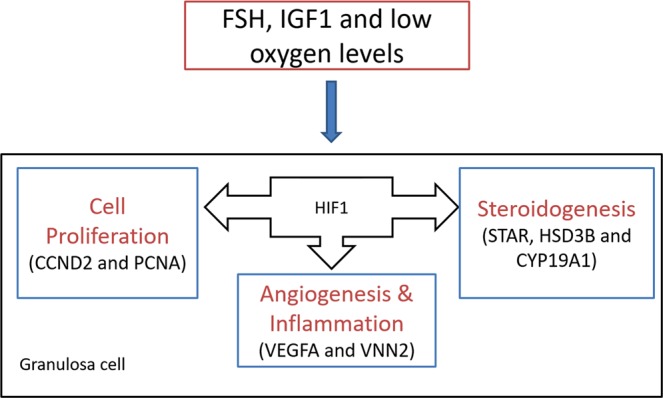
HIF1 is essential for granulosa cell function. Expression of HIF1A is induced by FSH, IGF1 and low oxygen levels in bovine granulosa cells. HIF1 may regulate steroidogenesis, cell proliferation, angiogenesis and inflammation processes in developing ovarian follicles.

Our results indicate that HIF1 plays an essential role in bovine follicular development as HIF1 driven transcriptional activity could play a modulatory role in steroidogenesis and cell proliferation of cultured bovine GC, whereby HIF1 seems to act differentially on some target genes under normoxic vs. hypoxic conditions. Estradiol and progesterone production may be differentially regulated by HIF1 under normoxic and hypoxic conditions, possibly due to the inhibition of FSH signaling under hypoxia. Overall, our data suggest that HIF1 driven transcriptional activity plays a crucial role in GC functionality in bovine ovarian follicles (Fig. 9).

## Materials and Methods

### Cell culture

Bovine ovarian samples used for obtaining the GC are collected from a local abattoir and does not require ethical permission according to the German law. The number of ovaries for each lot of collection (usually 8 to 12 ovaries) depends upon the availability of animals at the abattoir. Therefore the number of animals and ovaries used for the experiments is varied in general but assures the heterogeneous population of primary GC in each cell culture experiment. Ovaries were collected in ice-cold 1x PBS containing antimicrobials such as streptomycin (0.1 mg/ml), penicillin (100IU) and amphotericin (0.5 µg/µl). GC were aspirated from the ovaries with a syringe and 18 gauge needle and cryopreserved in freezing medium consisting of 90% fetal calf serum and 10% Dimethyl sulfoxide. For cell culture experiments, cryopreserved cells were thawed in a water bath, washed and re-suspended in α-MEM, and plated at a density of ~1.4 × 10^5^ viable cells, according to the trypan blue exclusion assay, in each well of 24 well culture dishes, which were pre-coated with 0.02% collagen R (Serva, Heidelberg). The α-MEM was reconstituted with 0.084% sodium bicarbonate, 0.1% BSA, 20 mM HEPES, 2 mM L-Glutamin, 4 ng/ml sodium selenite, 5 µg/ml transferrin, 10 ng/ml insulin, 1 mM non-essential amino acids, 100 IU penicillin and 0.1 mg/ml streptomycin. Additionally, 50 ng/ml IGFI (Sigma Aldrich, Steinheim, Germany), 20 ng/ml FSH (Folltropin-V, Vetoquinol; Sigma Aldrich, Steinheim, Germany), and 2 μM androstenedione (Sigma Aldrich, Steinheim, Germany) were added to the α-MEM before plating the cells. For identifying the effect of FSH and IGF1 on *HIF1A* mRNA expression under normoxia, culture media were prepared with either FSH (20 ng/ ml) or IGF1 (50 ng/ ml). For normoxic conditions, culture plates were maintained in a CO_2_ incubator at 21% O_2_ and 5% CO_2_ for 8 days while spent media was replaced every 2^nd^ day. For hypoxic conditions, a subset of culture plates was incubated at 1% O_2_ and 5% CO_2_ on day 6 for 48 hrs. α-MEM was supplemented with different concentrations of (1 nM, 3 nM and 5 nM) echinomycin on day 6 to inhibit *HIF1*. For *HIF1A* knockdown, GCs were transfected with 50 nM of antisense LNA negative control gapmer (A*A*C*A*C*-G*T*C*T*A*T*A*C* G*C), and anti *HIF1A* gapmer (A*C*T*G*A*T*C*G*A*A*G*G*A*-A*C*G) using TransIT-X2® transfection reagent (Mirus Bio, USA) as per the manufacturer’s recommendations in a 24 well culture dishes for 6 days (from day 2 to day 8 of the culture). As established before, all cultured cells were subjected to different analyses on day 8^20–23^.

### RNA isolation, cDNA synthesis, and quantitative real-time PCR analysis

Total RNA was isolated using innuPREP RNA Mini Kit (Analytik Jena, Germany) according to the manufacturer’s recommendations. The eluted RNA was quantified using a NanoDrop1000 Spectrophotometer (Thermo Scientific, Bonn, Germany). 200 ng total RNA was used for first-strand cDNA synthesis using SensiFAST cDNA Synthesis Kit (Bioline, Luckenwalde, Germany). Quantitative real-time PCR (qPCR) was performed using a light cycler 96 instrument (Roche, Mannheim, Germany). All PCR products amplified with the different primers (Supplementary Information: S1) were initially cloned in a pGEM-T vector (Promega biosciences, USA) and sequenced to verify primer pair specificity. If the product is correct, cloned vectors at five different serially diluted concentrations (5 × 10^−12^ to 5 × 10^−16^ g plasmid) were used for generating the standard curve in each run of qPCR. The abundance of transcripts was normalized using mean expression values of RPLP0 and TBP housekeeping transcripts. The melting curve analysis followed by agarose gel (3%) electrophoresis of PCR products was performed to ensure the correct identity of amplicons (Supplementary Information: S2).

### Capillary western blot

Western blots were executed using a WES instrument (Protein simple, CA, USA) according to the manufacturer’s guidelines. Briefly, cultured cells were lysed on ice using 1x RIPA buffer (Thermo Fischer, USA) followed by sonication and centrifugation steps to collect the protein supernatant. Protein concentrations in the supernatant were measured using BCA protein estimation assay kit (Thermo Fischer, USA). Protein samples (2 µg), wash buffers, blocking reagent, antibodies, and chemiluminescent substrate were prepared and distributed into the appropriate wells of assay plates. Subsequently, assay plates were loaded to the WES instrument, and proteins were allowed to separate. Detection of bands was performed automatically in the individual capillaries by the WES instrument. Anti HIF1A primary antibody (Novus biologicals, USA; Catalog# NBP1-02160SS; 1:250) and anti-beta actin primary antibody (Santa Cruz Biotechnology, Santa Cruz, CA, USA; Catalog No sc-47778: 1:250) were used together with anti-rabbit and anti-mouse secondary antibody supplied by ProteinSimple.

### Immunolocalization of HIF1α in ovarian follicles

Four bovine ovaries were obtained from a local abattoir and sliced into small pieces for fixation in Bouin’s reagent (10 ml of 37% formalin, 50 ml of glacial acetic acid and 150 ml of picric acid) for two days. Tissue samples were then dehydrated in a gradient series of ethanol and subjected to paraffin embedding using an MPS/W instrument (SLEE medical GmbH, Germany). 5 μm sections were prepared using a microtome and mounted into glass slides. Sections were deparaffinized and blocked with 2% BSA for 1 hr and incubated with either anti-HIF1A antibody (Invitrogen, Catalog No. MA1-16504; 1:100) in 1% BSA or 1% BSA (without antibody) as control overnight. Subsequently, slides were washed four times with wash buffer and incubated with secondary rabbit anti-mouse IgG(F(ab′)2) antibody (Sigma Aldrich, catalog No. AQ160B) for 1 hr. Slides were then treated with streptavidin HRP conjugate (Sigma, Catalog No. 1089153) for 40 min followed by incubation with HRP substrate, DAB, for 30 sec. All slides were counterstained with mayers hemalum solution for 10 sec. Finally, Roti mount aqua solution was added on each slide for color protection, and images were obtained using a bright-field with Axio imager A1 microscope (Carl Zeiss Inc, Germany).

### Cell viability assay

The spent media were collected in 1.5 ml tubes and centrifuged to pellet the floating dead cells. Adherent GC were washed twice with 1x PBS and detached using 250 µl of tryplE reagent per each well. The detached cells were merged with floating cells to ensure the inclusion of dead cell portions. Cells were washed with 1 ml α-MEM and resuspended in 100 µl of 1x binding buffer. 10 µl of Annexin V reagent was added to the cell suspension and incubated in the dark for 15 min followed by washing to remove the unbound dye and resuspension in 500 µl of 1x binding buffer. 5 µl of propidium iodide (500 µg/ml) was added to the cells just before flow cytometric analysis. The fluorescence signals from single cells were quantified by using a flow cytometer (Gallios, Beckman-Coulter) and the data were analyzed using the Kaluza software (Beckman-Coulter).

### Steroid hormone estimation

The concentrations of estradiol and progesterone in the spent media were measured through a competitive 3H–radioimmunoassay (RIA) using custom generated purified rabbit-raised antibodies. [2, 4, 6, 7-3 H] 17β-estradiol (GE Healthcare, Freiburg, Germany) and [1, 2, 6, 7-3 H (N)] progesterone (PerkinElmer, Boston, USA) were used as tracer molecules for E2 and P4 estimation, respectively. The minimum detection limit for E2 and P4 was 3 pg/ml and 7 pg/ml respectively with inter and intra assay coefficient of variation of 9.9% and 6.9% for estradiol, and 7.6% and 9.8% for progesterone. The standards were prepared by dissolving E2 and P4 in 100% ethanol which was subsequently diluted in RIA buffer. Spent media were diluted 1:40 in RIA buffer for P4 estimation and used undiluted for E2 estimation. All measurements were executed in duplicates. The radioactivity levels were analyzed in a liquid scintillation counter (TriCarb 2900 TR; PerkinElmer) with an integrated RIA-calculation program.

### Cell cycle assay

The number of cells in the proliferation was determined based on the detected DNA fluorescence in flow cytometric analysis. Cells were detached by adding 250 µl TryplE reagent (Thermo Fischer, USA) to each culture well. Subsequently, cells were washed and resuspended in 300 µl of 1x PBS. The cell suspension was dropwise added into 70% ice-cold ethanol and stored at −20 °C for 2 hr. Later, cells were pelleted at 300 g for 10 min and incubated in 1 ml of RNase solution (1 mg/ml) at 37 °C for 30 min. Propidium iodide reagent (50 µg/ml) was added to the cells and incubated in the dark at 37 °C for 30 min. The fluorescence signal was quantified from single cells (10,000 counts) using an EPICS-XL flow cytometer (Beckman-Coulter, Krefeld, Germany). Data were analyzed using the Multicycle software (Phoenix, USA).

### Chromatin immuno-precipitation (ChIP)

ChIP was performed using the MAGNA ChIP kit (Millipore) by following the manufacturer’s instructions. The cells were cultured in 60 cm^2^ culture plates at an initial seeding density of 40 × 10^5^ cells/plate in 12 ml of α-MEM, supplemented with 50 ng/ml IGF1 and 20 ng/ml FSH for 8 days while α-MEM was replaced every 2^nd^ day. On day 8, 1% formaldehyde was added to the cultured plates to crosslink the chromatin with proteins. After 10 min, cells were treated with 10x glycine for quenching the unreacted formaldehyde in the media. The cells were then washed twice with 1x PBS and collected into a 1.5 ml collecting tube using a cell scraper. The cells were then pelleted and dissolved in lysis buffer containing protease inhibitor cocktail (PIC). The cells in lysis buffer were sonicated using a Covaris S220 instrument according to the manufacturer’s recommendation. For immunoprecipitation, 450 µl of dilution buffer containing PIC was added to 50 µl of sonicated DNA. From this, 5 µl was aliquoted for input DNA analysis and stored at 4 °C. 5 µg of anti-HIF1A antibody (Invitrogen, Catalog #. MA1-16504) and 20 µl of fully suspended protein AG beads were added to the remaining solution followed by incubation overnight on a rotator at 4 °C. Unspecific mouse Ig G (Merck Millipore) and specific anti-RNA polymerase antibody (Merck Millipore) were used as a negative and positive control, respectively. On the next day, chromatin-antibody-magnetic bead complexes were washed with a series of wash buffers such as low salt, high salt, lithium chloride and Tris EDTA buffer, provided in the Magna ChIP kit. All protein-DNA complexes and inputs were subjected to reverse cross-linking by adding elution buffer containing proteinase K and incubated for 2 hr at 62 °C. DNA was eluted using silica-based polypropylene spin columns supplied in the MAGNA kit. The anti *HIF1A* antibody precipitated DNA was subjected to amplification of the proximal promoter (P2) region of *CYP19A1*^56,57^ together with positive and negative controls. SYBR green chemistry was used for the quantification of HIF1 binding. Percent input and ddCt methods were used to quantify the relative amount of HIF1A binding events on *CYP19A1* promotor.

### Statistical analysis

Analyses in the present study were executed from different cell preparations with three independent primary granulosa culture experiments. Untransformed data values have been used for data analysis. Statistical interpretations were derived using Graphpad Prism software. Data were analyzed using one-way repeated measures analysis of variance. Pairwise multiple comparisons were executed using post hoc Tukey’s test. Student t-test was applied for the comparisons between two groups (e.g., control gapmer Vs. HIF1A gapmer). Data is presented as MEAN ± SEM values in all figures and tables. Probability (p) values  < 0.05 were considered as statistically significant and are designated with up to three asterisk symbols to inform the strength of significant difference (*p < 0.05; **p < 0.005; ***p < 0.0005).

## Supplementary information

Supplementary Information.

## Data Availability

All the data and materials of the present study have been presented in the manuscript and in the Supplementary Information.

## References

[1] Graham AM, Presnell JS (2017). Hypoxia Inducible Factor (HIF) transcription factor family expansion, diversification, divergence and selection in eukaryotes. PloS one.

[2] Wang GL, Jiang B-H, Rue EA, Semenza GL (1995). Hypoxia-inducible factor 1 is a basic-helix-loop-helix-PAS heterodimer regulated by cellular O2 tension. Proceedings of the national academy of sciences.

[3] Maxwell PH (1999). The tumour suppressor protein VHL targets hypoxia-inducible factors for oxygen-dependent proteolysis. Nature.

[4] Majmundar AJ, Wong WJ, Simon MC (2010). Hypoxia-inducible factors and the response to hypoxic stress. Molecular cell.

[5] Wang Z (2011). Contribution of hypoxia inducible factor-1α to the profibrotic action of angiotensin II in cultured renal medullary interstitial cells. Kidney international.

[6] Fukuda R, Kelly B, Semenza GL (2003). Vascular endothelial growth factor gene expression in colon cancer cells exposed to prostaglandin E2 is mediated by hypoxia-inducible factor 1. Cancer research.

[7] Gerber SA, Pober JS (2008). IFN-α induces transcription of hypoxia-inducible factor-1α to inhibit proliferation of human endothelial cells. The Journal of Immunology.

[8] Fukuda R (2002). Insulin-like growth factor 1 induces hypoxia-inducible factor 1-mediated vascular endothelial growth factor expression, which is dependent on MAP kinase and phosphatidylinositol 3-kinase signaling in colon cancer cells. Journal of Biological Chemistry.

[9] Vallée A, Guillevin R, Vallée J-N (2017). Vasculogenesis and angiogenesis initiation under normoxic conditions through Wnt/β-catenin pathway in gliomas. Reviews in the Neurosciences.

[10] Zhang J (2018). Initiation of follicular atresia: gene networks during early atresia in pig ovaries. Reproduction.

[11] Henríquez S (2017). *In-vitro* study of gonadotrophin signaling pathways in human granulosa cells in relation to progesterone receptor expression. Reproductive biomedicine online.

[12] Kim J, Bagchi IC, Bagchi MK (2009). Signaling by hypoxia-inducible factors is critical for ovulation in mice. Endocrinology.

[13] Zhang Z (2015). Expression of hypoxia-inducible factor-1α during ovarian follicular growth and development in Sprague-Dawley rats. Genet. Mol. Res.

[14] Boonyaprakob U, Gadsby JE, Hedgpeth V, Routh PA, Almond GW (2005). Expression and localization of hypoxia inducible factor-1alpha mRNA in the porcine ovary. Can. J. Vet. Res..

[15] Berisha B, Schams D, Rodler D, Sinowatz F, Pfaffl M (2017). Expression pattern of HIF 1alpha and vasohibins during follicle maturation and corpus luteum function in the bovine ovary. Reproduction in Domestic Animals.

[16] Alam H (2004). Follicle-stimulating hormone activation of hypoxia-inducible factor-1 by the phosphatidylinositol 3-kinase/AKT/Ras homolog enriched in brain (Rheb)/mammalian target of rapamycin (mTOR) pathway is necessary for induction of select protein markers of follicular differentiation. Journal of Biological Chemistry.

[17] Yalu R, Oyesiji AE, Eisenberg I, Imbar T, Meidan R (2015). HIF1A-dependent increase in endothelin 2 levels in granulosa cells: role of hypoxia, LH/cAMP, and reactive oxygen species. Reproduction.

[18] Kowalewski MP, Gram A, Boos A (2015). The role of hypoxia and HIF1α in the regulation of STAR-mediated steroidogenesis in granulosa cells. Molecular and cellular endocrinology.

[19] Thompson JG, Brown HM, Kind KL, Russell DL (2015). The Ovarian Antral Follicle: Living on the Edge of Hypoxia or Not?. Biol. Reprod.

[20] Baufeld A, Vanselow J (2013). Increasing cell plating density mimics an early post-LH stage in cultured bovine granulosa cells. Cell Tissue Res.

[21] Yenuganti Vengala Rao, Vanselow Jens (2017). Cultured bovine granulosa cells rapidly lose important features of their identity and functionality but partially recover under long-term culture conditions. Cell and Tissue Research.

[22] Baddela, V. S., Sharma, A., Viergutz, T., Koczan, D. & Vanselow, J. Low oxygen levels induce early luteinization associated changes in bovine granulosa cells. *Frontiers in physiology* **9** (2018). 10.3389/fphys.2018.01066 PMC6090175 30131718

[23] Sharma, A. *et al*. Elevated free fatty acids affect bovine granulosa cell function: a molecular cue for compromised reproduction during negative energy balance. *Endocrine connections* **1** (2019). 10.1530/EC-19-0011 PMC6479201 30925464

[24] Samarajeewa NU (2013). HIF-1alpha stimulates aromatase expression driven by prostaglandin E2 in breast adipose stroma. Breast Cancer Res..

[25] Behl, R. & Kaul, R. Insulin like growth factor 1 and regulation of ovarian function in mammals (2002). 12561963

[26] Howles CM (2000). Role of LH and FSH in ovarian function. Molecular and cellular endocrinology.

[27] Baufeld, A. & Vanselow, J. A Tissue Culture Model of Estrogen-producing Primary Bovine Granulosa Cells. *Journal of visualized experiments: JoVE* (2018). 10.3791/58208 PMC6235104 30247464

[28] Baufeld A, Vanselow J (2013). Increasing plating density mimics LH-induced gene expression patterns in cultured bovine granulosa cells. Reproductive Biology.

[29] Yenuganti, V. R., Baddela, V. S., Baufeld, A., Singh, D. & Vanselow, J. The gene expression pattern induced by high plating density in cultured bovine and buffalo granulosa cells might be regulated by specific miRNA species. *Journal of Reproduction and Development*, 2014–2119 (2015). 10.1262/jrd.2014-119 PMC4410314 25740097

[30] Baddela VS, Onteru SK, Singh D (2017). A syntenic locus on buffalo chromosome 20: novel genomic hotspot for miRNAs involved in follicular-luteal transition. Functional & integrative genomics.

[31] Yadav M, Agrawal H, Pandey M, Singh D, Onteru SK (2018). Three-dimensional culture of buffalo granulosa cells in hanging drop mimics the preovulatory follicle stage. Journal of cellular physiology.

[32] Ma L (2019). Hypoxia Limits the Growth of Bovine Follicles *in Vitro* by Inhibiting Estrogen Receptor α. Animals.

[33] Byun Y (2019). MiR-200c downregulates HIF-1α and inhibits migration of lung cancer cells. Cellular & molecular biology letters.

[34] Li L (2017). lncRNAs HIF1A-AS2 facilitates the up-regulation of HIF-1α by sponging to miR-153-3p, whereby promoting angiogenesis in HUVECs in hypoxia. Biomedicine & Pharmacotherapy.

[35] Bartoszewska S (2015). The hypoxia-inducible miR-429 regulates hypoxia-inducible factor-1 α expression in human endothelial cells through a negative feedback loop. The FASEB Journal.

[36] Han F, Wu Y, Jiang W (2015). MicroRNA-18a decreases choroidal endothelial cell proliferation and migration by inhibiting HIF1A expression. Medical science monitor: international medical journal of experimental and clinical research.

[37] Shen G, Li X, Jia Y-f, Piazza GA, Xi Y (2013). Hypoxia-regulated microRNAs in human cancer. Acta Pharmacologica Sinica.

[38] Chua YL (2010). Stabilization of hypoxia-inducible factor-1α protein in hypoxia occurs independently of mitochondrial reactive oxygen species production. Journal of Biological Chemistry.

[39] Rico C (2014). HIF1 activity in granulosa cells is required for FSH-regulated Vegfa expression and follicle survival in mice. Biology of reproduction.

[40] Jośko J, Mazurek M (2004). Transcription factors having impact on vascular endothelial growth factor (VEGF) gene expression in angiogenesis. Medical Science Monitor.

[41] Sayasith K, Sirois J, Lussier JG (2013). Expression, regulation, and promoter activation of Vanin-2 (VNN2) in bovine follicles prior to ovulation. Biol. Reprod..

[42] Christenson LK (2013). Research resource: preovulatory LH surge effects on follicular theca and granulosa transcriptomes. Mol. Endocrinol..

[43] Biddlestone J, Bandarra D, Rocha S (2015). The role of hypoxia in inflammatory disease. International journal of molecular medicine.

[44] Andersen CY, Ezcurra D (2014). Human steroidogenesis: implications for controlled ovarian stimulation with exogenous gonadotropins. Reproductive Biology and Endocrinology.

[45] Wang, X. *et al*. HIF 1 inhibits STAR transcription and testosterone synthesis in murine Leydig cells. *Journal of molecular endocrinology* **1** (2018). 10.1530/JME-18-0148 30400066

[46] Lysiak JJ (2009). Hypoxia-inducible factor-1α is constitutively expressed in murine Leydig cells and regulates 3β-hydroxysteroid dehydrogenase type 1 promoter activity. Journal of andrology.

[47] Sousa L (2016). Is the canine corpus luteum an insulin-sensitive tissue?. The Journal of endocrinology.

[48] Yu RMK (2015). Evidence for microRNA-mediated regulation of steroidogenesis by hypoxia. Environmental science & technology.

[49] Fadhillah, Yoshioka S, Nishimura R, Okuda K (2014). Hypoxia promotes progesterone synthesis during luteinization in bovine granulosa cells. J. Reprod. Dev..

[50] Yenuganti VR, Viergutz T, Vanselow J (2016). Oleic acid induces specific alterations in the morphology, gene expression and steroid hormone production of cultured bovine granulosa cells. Gen. Comp. Endocrinol..

[51] Douville G, Sirard M-A (2014). Changes in granulosa cells gene expression associated with growth, plateau and atretic phases in medium bovine follicles. Journal of ovarian research.

[52] Goda N (2003). Hypoxia-inducible factor 1α is essential for cell cycle arrest during hypoxia. Molecular and cellular biology.

[53] Hubbi ME, Semenza GL (2015). Regulation of cell proliferation by hypoxia-inducible factors. American Journal of Physiology-Cell Physiology.

[54] Yonekura S (2013). Effects of the HIF1 inhibitor, echinomycin, on growth and NOTCH signalling in leukaemia cells. Anticancer research.

[55] Ortmann B, Druker J, Rocha S (2014). Cell cycle progression in response to oxygen levels. Cellular and molecular life sciences.

[56] Vanselow J (2001). Expression of the aromatase cytochrome P450 encoding gene in cattle and sheep. Journal of Steroid Biochemistry and Molecular Biology.

[57] Fürbass R, Kalbe C, Vanselow J (1997). Tissue-specific expression of the bovine aromatase-encoding gene uses multiple transcriptional start sites and alternative first exons. Endocrinology.

