# Supplementary material for: HIF1 driven transcriptional activity regulates steroidogenesis and proliferation of bovine granulosa cells
Source: Sci Rep. 2020 Mar 3;10:3906. doi: 10.1038/s41598-020-60935-1 (PMC7054295; doi:10.1038/s41598-020-60935-1)
Supplement: Supplementary file 1 — Supplementary Information. [file 41598_2020_60935_MOESM1_ESM.pdf]

# Supplementary information

## HIF1 driven transcriptional activity regulates steroidogenesis and proliferation of bovine granulosa cells

**Authors**: Vijay Simha Baddela[1], Arpna Sharma[1], Marten Michaelis[2], Jens Vanselow[1]*

**Affiliations**: [1] Experimental Reproductive Biology Unit, Institute of Reproductive Biology, Leibniz Institute for Farm Animal Biology (FBN), 18196, Dummerstorf, Germany; [2] Reproductive Biochemistry Unit, Institute of Reproductive Biology, Leibniz Institute for Farm Animal Biology (FBN), 18196 Dummerstorf, Germany

**Correspondence**: Jens Vanselow, Experimental Reproductive Biology Unit, Institute of Reproductive Biology, Leibniz Institute for Farm Animal Biology (FBN), Wilhelm-Stahl-Allee 2, 18196 Dummerstorf, Germany; Tel: +49 38208 68750; Fax: +49 38208 68752;
e-mail: vanselow@fbn-dummerstorf.de

**S1: PCR primers:**

| Gene | | Sequence | Accession No. | Product Length |
|---|---|---|---|---|
| RPLP0 | For | TGGTTACCCAACCGTCGCATCTGTA | NM_001012682 | 142 bp |
| | Rev | CACAAAGGCAGATGGATCAGCCAAG | | |
| TBP | For | GCCTTGTGCTTACCCACCAACAGTTC | NM_001034494 | 200 bp |
| | Rev | TGTCTTCCTGAAACCCTTCAGAATAGGG | | |
| VEGFA | For | AGGAGCCTCCCTCAGGGTTTCGGG | NM_001075742 | 183 bp |
| | Rev | CGCTCCGGACCCAAAGTGCTCTGC | | |
| HIF1A | For | GCATCGCGGGCACCGATTCACCAT | NM_174339.3 | 165 bp |
| | Rev | TGGGGGAGTGGCAACTGATGAGCA | | |
| STAR | For | TTGTGAGCGTACGCTGTACCAAG | NM_174189.2 | 237 bp |
| | Rev | CTGCGAGAGGACCTGGTTGATG | | |
| CYP19A1 | For | GCTTTTGGAAGTGCTGAACCCAAGG | NM_174305 | 172 bp |
| | Rev | GGGCCCAATTCCCAGAAAGTAGCTG | | |
| CYP19A1 | For | CCACCTCTGGAATGAGCTGC | NM_174305 | 272 bp |
| (ChIP) | Rev | GTACCGTAGAGATTGGCGCTT | | |
| HSD3B | For | TGTTGGTGGAGGAGAAGGATCTG | NM_174343 | 208 bp |
| | Rev | GCATTCCTGACGTCAATGACAGAG | | |
| CCND2 | For | CGCAGGGCCGTGCCGGACGCCAAC | NM_001076372 | 279 bp |
| | Rev | CACGGCCCCCAGCAGCTGCAGATGG | | |
| PCNA | For | GTGAACCTGCAGAGCATGGACTCGT | NM_001034494 | 192 bp |
| | Rev | CGTGTCCGCGTTATCTTCAGCTCTT | | |
| VNN2 | For | TCCCACAGCTTGGATGAACGTTTTG | NM_001163920 | 267 bp |
| | Rev | TAGGCACTCCAATTCATGGCTGGTG | | |

**S2: PCR product specificity of different genes under the study:**

All the PCR primers used in the study were verified for single product amplification using 3% agarose gel electrophoresis after each run. The gel pictures below indicate a positive control (standard) and negative control (n.k) samples. The negative control well contains nuclease pure water instead of DNA.

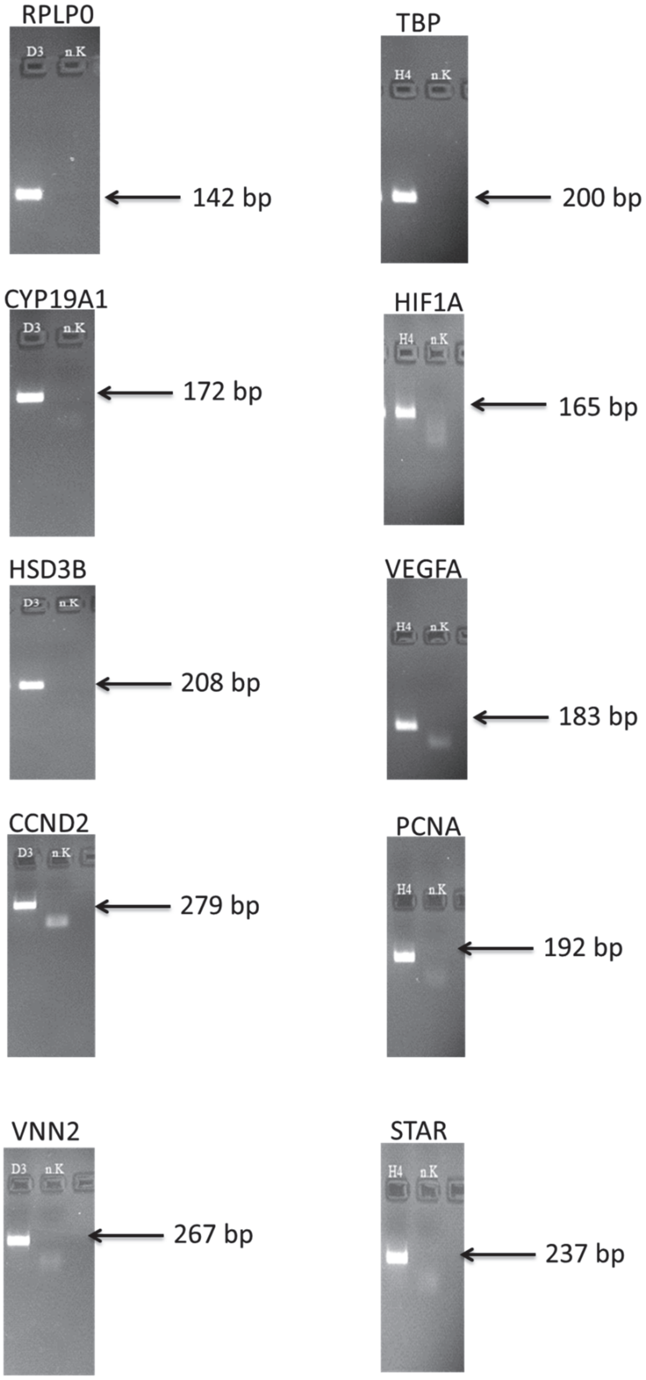

**S3: Morphology of Granulosa cells after treating with echinomycin under normoxic and hypoxic conditions:**

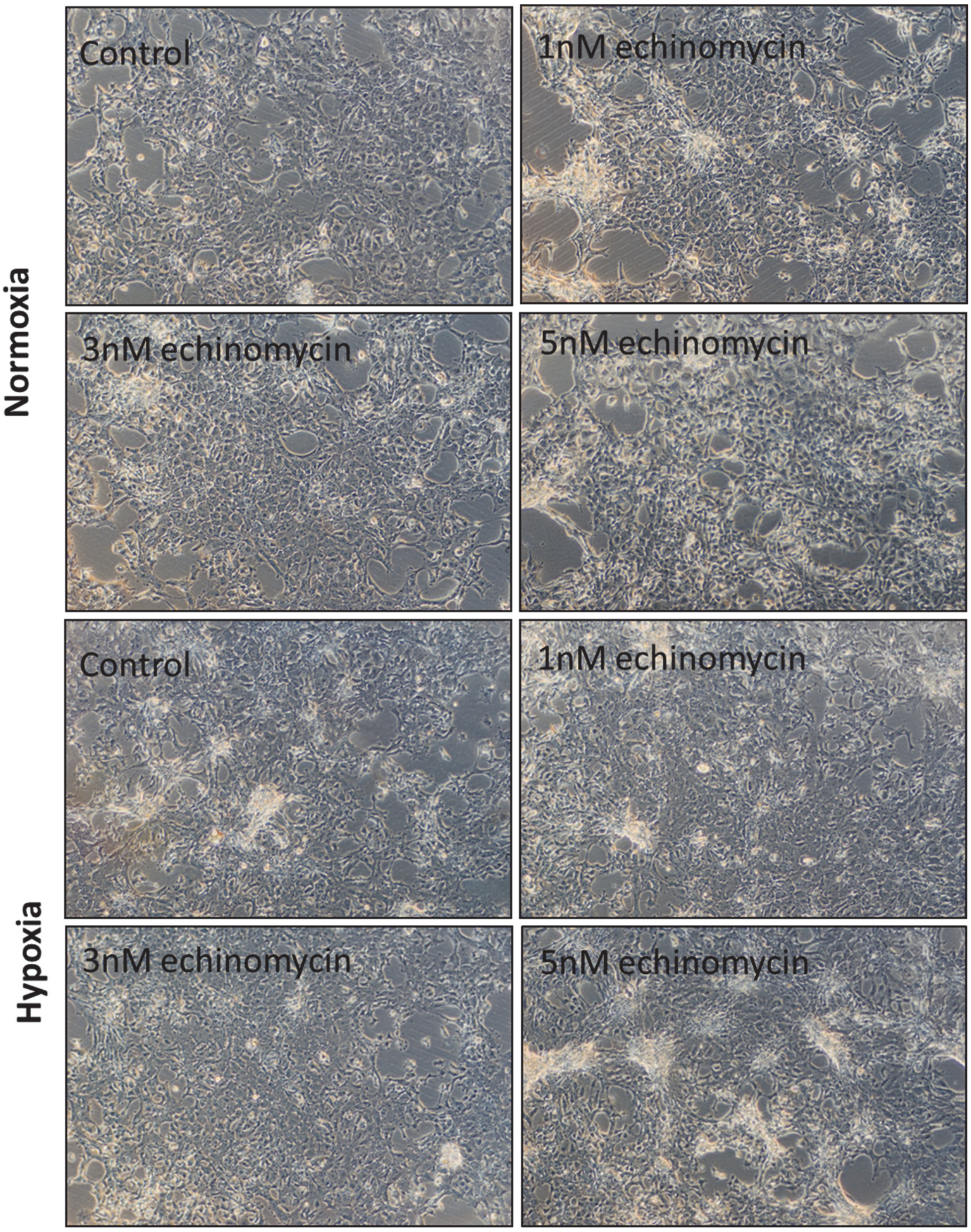


Photomicrographs of GC cultured under serum-free culture system for 8 days. On 6th day GC were treated with different concentrations of echinomycin. Photomicrographs were obtained from Nikon TMS-F inverted microscope with 200X magnification.

## S4: Cell viability status in granulosa cells after treatment with echinomycin:

| | 21% Oxygen | | | 1% Oxygen | | |
|---|---|---|---|---|---|---|
| (%) | Viable cells | Apoptotic cells | Dead cells | Viable cells | Apoptotic cells (%) | Dead cells (%) |
| Control | 78.167±2.64 | 15.37±1.83 | 5.62±0.67 | 78.65±4.67 | 15.76±3.79 | 5.137±0.96 |
| 5nM Echinomycin | 79.160±5.43 | 9.49±1.46 | 10.49±4.27 | 81.70± 4.25 | 11.72±2.84 | 6.013±11.36 |

- Representative histograms of FACS analysis used for identifying the cell viability and apoptotic status is below.

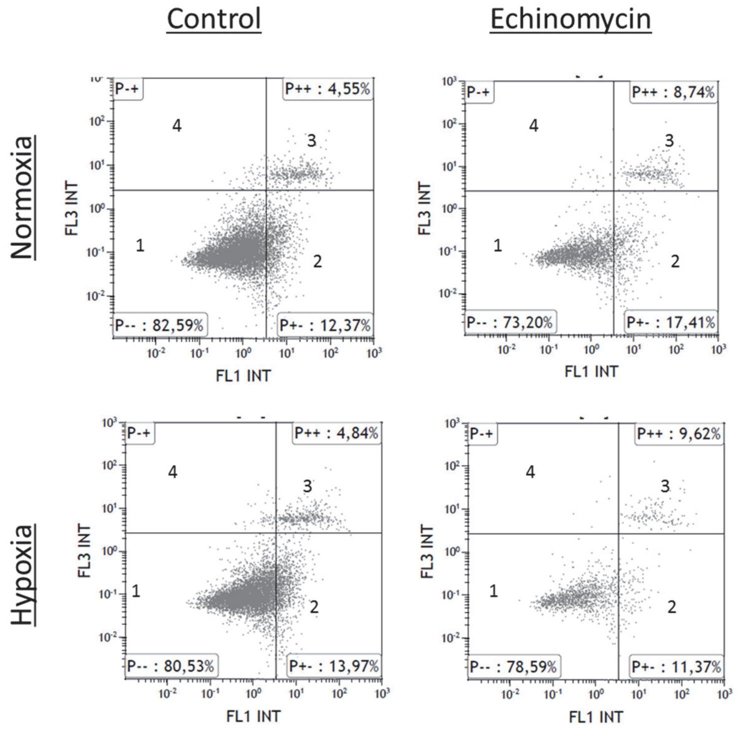


1: viable cells, 2: apoptotic, 3: dead, 4: necrotic cell proportions in the FACS analysis.

**S5: Morphology of Granulosa cells after treating with control and HIF1A gapmers:**

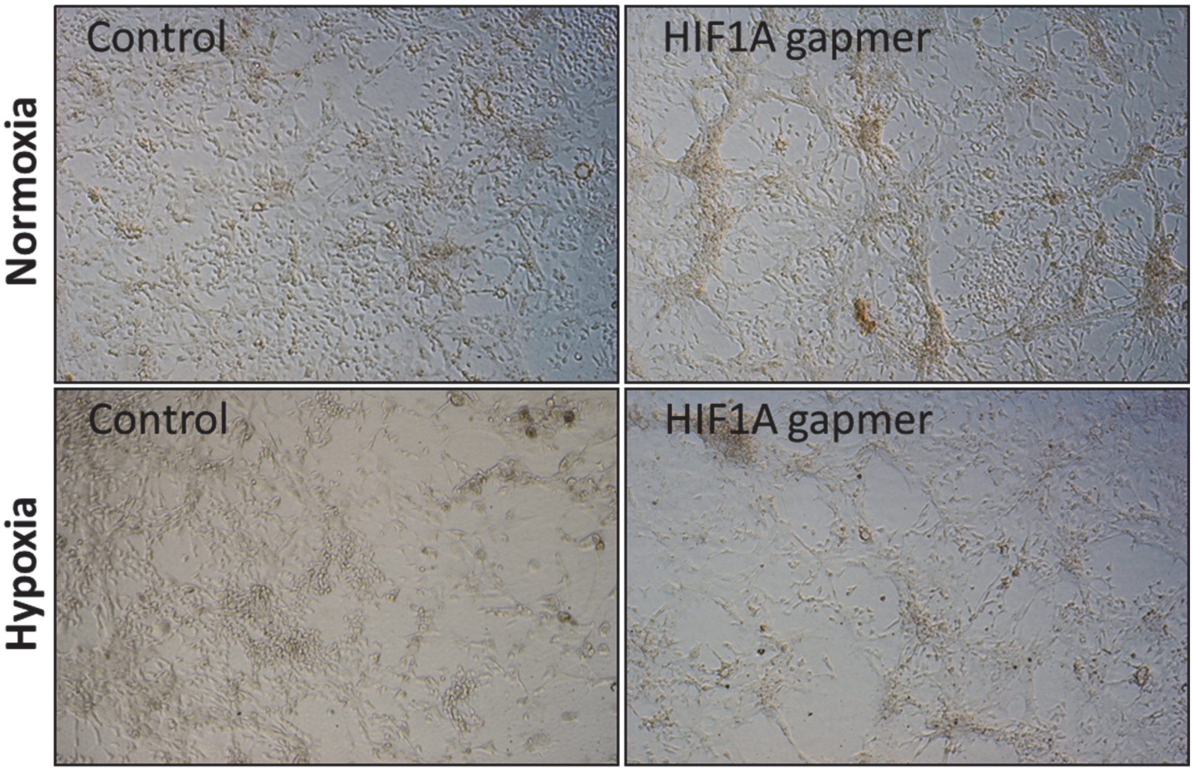


Photomicrographs of GC cultured under serum-free culture system for 8 days. On day 2, GC were incubated with 50nM of HIF1A gapmer for 6 days. Photomicrographs were obtained from Nikon TMS-F inverted microscope with 200X magnification.

**S6: Cell viability status in granulosa cells after treating with control and HIF1A gapmers:**

| | 21% Oxygen | | | 1% Oxygen | | |
|---|---|---|---|---|---|---|
| (%) | Viable cells | Apoptotic cells | Dead cells | Viable cells | Apoptotic cells (%) | Dead cells (%) |
| Control Gapmer | 91.5±1.5 | 5.6±1.0 | 2.4±0.4 | 90.1±1.9 | 5.3±1.5 | 3.9±0.3 |
| HIF1A Gapmer | 92.0±0.8 | 5.8±0.7 | 1.9±0.1 | 89.5±2.0 | 7.1±1.7 | 2.8±0.3 |

- Representative histograms of FACS analysis used for identifying the cell viability and apoptotic status is below

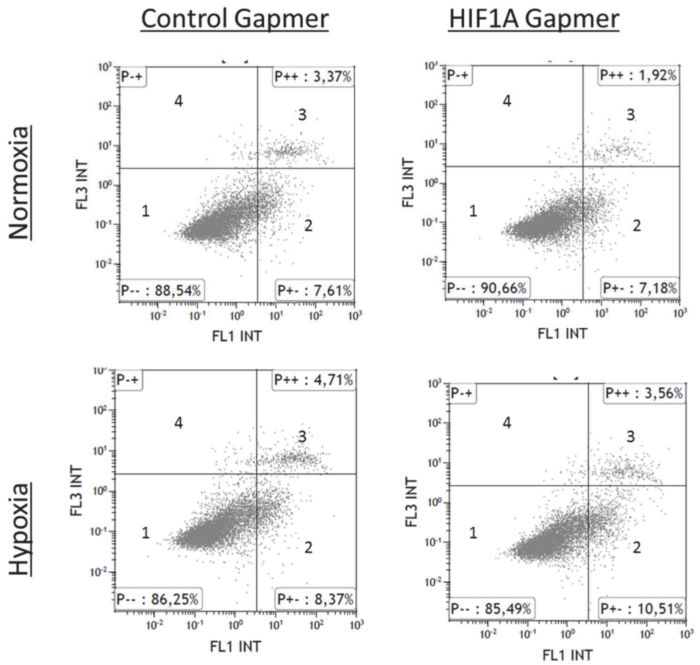


1: viable cells, 2: apoptotic, 3: dead, 4: necrotic cell proportions in the FACS analysis.
